# Morphology of the Spleen in *Oreochromis niloticus*: Splenic Subregions and the Blood-Spleen Barrier

**DOI:** 10.3390/ani11102934

**Published:** 2021-10-11

**Authors:** Yang He, Erlong Wang, Kaiyu Wang, Jun Wang, Wei Fan, Defang Chen, Qian Yang

**Affiliations:** 1 Key Laboratory of Animal Disease and Human Health of Sichuan Province, Department of Basic Veterinary, College of Veterinary Medicine, Sichuan Agricultural University, Wenjiang 611130, China; 2 Key Laboratory of Sichuan Province for Fishes Conservation and Utilization in the Upper Reaches of the Yangtze River, Neijiang Normal University, Neijiang 641000, China; wangjun1986616@gmail.com; 3 College of Animal Science and Technology, Northwest A&F University, Xianyang 712100, China; welsicau@126.com; 4 Neijiang City Academy of Agricultural Sciences, Neijiang 641000, China; fanwey1990@outlook.com; 5 Department of Aquaculture, College of Animal Science & Technology, Sichuan Agricultural University, Wenjiang 611130, China; chendf_sicau@126.com; 6 Laboratory Animal Center, Southwest Medical University, Longmatan District Xianglin Road No.1, Luzhou 646000, China; yangqiansicau@126.com

**Keywords:** Nile tilapia, spleen, subregion, blood-spleen barrier

## Abstract

**Simple Summary:**

The spleen is a separate organ of the teleost, playing an essential role in immune reactions. The morphology of the spleen is different from the fish species. Little knowledge about the spleen structure and the blood splenic barrier (BSB) in Nile tilapia has been reported. The present study showed that the spleen of Nile tilapia could be portioned into three subregions, and the BSB lay in the middle layer, composed of the cuboidal-shaped endotheliocytes and the surrounding reticular fibers of the ellipsoid capillaries. Our results enriched the research of immune tissues and system in tilapia and provided reference for the study of spleen in other fish species.

**Abstract:**

The spleen is a separate organ of the teleost, playing an essential role in immune reactions. The morphology of the spleen is different from the fish species. Little knowledge about the spleen structure and the blood splenic barrier (BSB) in Nile tilapia has been reported. To address this issue, we studied the histology of the spleen and the BSB in healthy Nile tilapia. The morphology of the spleen was observed, then H&E staining, modified Jame’s staining, and ultrastructural techniques were performed to portion the spleen into three subregions and analyze the location of components and fibers. Thereafter, vital staining of Nile tilapia with Trypan blue was conducted to elucidate the composition and function of BSB. Histologically, the spleen could be divided into three subregions (inner, middle, and outer). The venules, clumps of lymphocytes, and vessels were separately characterized features of the outer, middle, and inner layers. Post injection, Trypan blue was intercepted in the endotheliocytes of ellipsoids in the middle layer (*i.p.*) or was deposited to the reticular fibers surrounding the ellipsoids (*i.v.*). Additionally, the amount of Trypan blue was shown to be positively correlated to that of the Acid phosphatase expressed. In conclusion, the spleen could be portioned into three subregions, and the BSB lay in the middle layer, composed of the cuboidal-shaped endotheliocytes and the surrounding reticular fibers of the ellipsoid capillaries. The present study enriched the research of immune tissues and system in tilapia and provided reference for the study of spleen in other fish species.

## 1. Introduction

It is necessary to improve the knowledge on fish immune organs since they are closely related to the immune responses. The detection of immune responses is a useful tool to reflect the fish health and disease status. Therefore, techniques for investigating immune responses have been widely used, such as RT-qPCR for the expression of immune genes, immunohistochemistry (IHC) or ELISA for immune proteins, and flow cytometric method for immune cells [1,2,3]. The histological technique is a gold standard widely used in human diagnosis and prognosis [4]. However, its application in teleost was limited due to the differences of morphology and histology among species [5]. 

The spleen, as one of the most important immune organs of teleost, plays essential roles in filtering and removing foreign agents or effete blood cells and secreting antibodies. Although the components of the teleost spleen are similar, the splenic morphology and histology vary greatly among species [5]. The blood spleen barrier (BSB), a biological barrier, is a vital component in splenic immune. It was first reported by Weiss in 1986 in murine [6], and its function found in stabilizing the microenvironment of white pulps and presenting antigen information to the white pulp [7]. The location of the BSB is found in the marginal zone in mammals [8] and in the ellipsoids and the periellipsoidal lymphatic sheaths (PELS) in species that lack marginal zones such as chicken [9], duck [10], and turtle [11]. 

As an important economic fish, Tilapia has been threatened by various pathogens for years [12,13]. Significant achievements have been made in diagnosis [14,15], vaccines [16,17], and pathogenesis [18,19]. However, disease outbreaks are reported frequently, which urges the essential exploration of new strategies for anti-infection [20]. The host-directed immunomodulatory is one of the promising approaches guided by the well-studied immune system. However, to obtain a greater knowledge of the immune system of this species, it is advantageous to investigate the histology and BSB of the spleen in Nile tilapia. We have performed the microstructural and ultrastructural methods to analyze the structure and components of the spleen. By applying vital staining with Trypan blue, we have elucidated if BSB exists in the spleen of Nile tilapia and participates in immune reactions.

## 2. Materials and Methods

### 2.1. Animal Ethics

Animal experiments were approved by the Animal Experiment Ethics Committee of Sichuan Agricultural University, Wenjiang, China (License No. XYF2020-028). Sixty healthy Nile tilapia (250 ± 20 g) were purchased from Pearl River Fisheries Research Institute of China (Guangdong, China) on the basis of an external examination for any signs of abnormalities or infestation. To avoid the anthropogenic effect as highlighted by Parrino V. et al. [21], the fish were acclimatized in concrete culture tanks (1.5 m × 1.0 m × 1.5 m) and fed with a commercial diet (Unif, Zhongshan, China) twice a day at 9:00 and 16:00 for two weeks in a flowing water system before experiments [22]. Fish were anesthetized by immersing with MS-222 (Sigma, St. Louis, MO, USA) before sampling.

### 2.2. Anatomy and Corrosion

Six fish were euthanized for analyzing the morphology of the spleen. Three fish were dissected from the left side of the body, and the others were examined from the right side. Dissections were started near the anus, cutting diagonally upward along the lateral line to the pectoral, whereafter downward to the pericardial cavity to unveil the entire abdominal cavity (Figure S1). The shape, color, and external vasculature of the spleen were recorded. Thereafter, all spleens were carefully sampled and immersed in ten volumes of 1% NaOH at room temperature until the internal vessels of the spleen were visible (about 20–30 days).

### 2.3. Microscopic Observation

Six fish were euthanized, and the spleens were collected post-mortem immediately. Three spleens were separated into two parts along the long axis, and the other three spleens were crosscut into three parts (named head, body, and tail). All samples were fixed in 4% paraformaldehyde (pH 7.2) for 24–48 h, dehydrated with a series upgrade ethanol, embedded in paraffin, and sliced (3 μm thick/section) for further staining. 

(a) Hematoxylin and eosin staining (H&E). Sections were stained with hematoxylin/eosin (H&E) according to Michalezykwetula [23]. The slides were observed using light microscopy (CX 33, Olympus, Japan). The splenic components and their locations were recorded. The thickness of the capsule was measured using the plugins of Measure lengths and distances Image-Pro Plus software version 6.0 (IPP 6.0) (Media Cybernetics, Inc., Bethesda, MD, USA).

(b) Jame’s staining. A modified Jame’s staining was conducted as described previously with minor modifications [24]. Briefly, after deparaffinization and rehydration, slides were oxidized with acidic KMnO_4_ (25 mL 0.3% KMnO_4_ and 25 mL 0.3% H_2_SO_4_) for 5 min, bleached with 5% oxalic acid for 5 min, stained with 5% silver nitrate (5 min), ammoniacal silver (5 min) and 5% formalin (5 min), respectively. Then, the slides were counterstained with nuclear fast red (5 min). After each step, slides were washed three times with ddH_2_O (5 min). Slides were dehydrated, cleared and mounted in resin before microscopic analysis (Nikon, Tokyo, Japan). The locations of collagen fibers and reticular fibers were recorded. The area ratio of reticular and collagen fibers was calculated in a standard area of 275,800 μm^2^ per field (×200 magnifications) using the Count and measure objects (area) of IPP 6.0 software.

### 2.4. Ultrastructural Observation

Samples were fixed in 2.5% cold glutaraldehyde, post-fixed in 1% osmium tetraoxide, dehydrated in gradient acetone baths (30%, 50%, 70%, 80%, 90%, 100% and 100%), then infiltrated in 1:4 acetone: epon resin for 8 h. After two hours of infiltration in pure epon resin, samples were embedded in resin, sectioned into 50 nm thick slices and stained with uranyl acetate and lead citrate. The ultrastructural structure was observed using a transmission electron microscope (TEM) (H600, Hitachi, Japan). 

For SEM analysis, a spleen was crosscut and dehydrated in gradient alcohol baths (25%, 50%, 75%, 95%, and 100%) and isoamyl acetate, respectively, followed by desiccation with carbon dioxide. The specimens were observed after being coated with gold on a rotatory stage [25] using a scanning electron microscope (SEM) (JSM-7500F, JEOL, Japan).

### 2.5. Vital Staining

First, 1 g Trypan blue was dissolved in 100 mL 0.85% saline, boiled for 10 min, and filtered with filter paper before use. A total of 27 fish were randomly divided into nine groups (three fish per group). Fish of four groups were injected with Trypan blue (0.1 mL/fish) intraperitoneally (*i.p.*). One group of fish were sampled for collecting of spleens every 4 h post-injection (hpi) until 72 hpi, respectively (4, 8, 24, and 72 hpi). Fish of the other four groups were injected with Trypan blue (0.1 mL/fish) intravenously, and spleens were collected at 1, 2, 3, and 24 hpi. The last group was used as a control group and fish were sampled before injection (0 hpi). Specimens were named after the sampling time plus injection methods (time + *i.p./i.v.*). Dynamic analyses of Trypan blue, ACP (Acid phosphatase), and histopathological changes were performed as described below. 

The spleens of fish in *i.p.* groups were separated into two parts along the long axis. One part was fixed in 4% paraformaldehyde, dehydrated, cleared, blocked by paraffin, and sliced (3 μm) for two continuous sections (one stained with H&E, one did not stain). The other part was stored at −80 ℃, imbedded with OCT, sliced (5 μm), stained with Gomori staining, and analyzed for kinetics of ACP. The spleens of fish in *i.v.* groups were separated as described above, but only one part was paraffined and sliced for two continuous sections, for H&E staining or not. The sections were stained by H&E and observed for Trypan blue location with Leica CD300 (Nikon, Japan). The slides without staining were analyzed for Area and IOD analysis of Trypan blue. Additionally, slides stained with Gomori staining were used for Area and IOD analysis of ACP. 

Briefly, five photos per slide were captured under a magnificent of 200×, whereafter the areas and IOD were recorded in each picture using the Count and measure objects (area) of IPP 6.0. Area and IOD data were normalized by dividing the data by a standard area (275,800 μm^2^). 

### 2.6. Statistical Analysis

Image-Pro Plus software version 6.0 (IPP 6.0) (Media Cybernetics, Inc.) was used for analysis. The thickness of the capsule was measured using the plugins of Measure lengths and distances. Areas or Integrated Optical Density (IOD) were measured using the plugin Count/Size. Five photos per section were captured under 200× magnification (a standard area of 275,800 μm^2^ per field). Data of areas and IOD were normalized by dividing a standard area (275,800), whereafter statistical analysis was performed using Graphpad prism 8.0.2 by one-way ANOVA with Dunnett’s multiple comparisons test. Data were expressed as mean ± SD. Values of *p* < 0.05 were considered statistically significant. 

## 3. Results

### 3.1. Macromorphology of Tilapia Spleen

**Figure 1:**
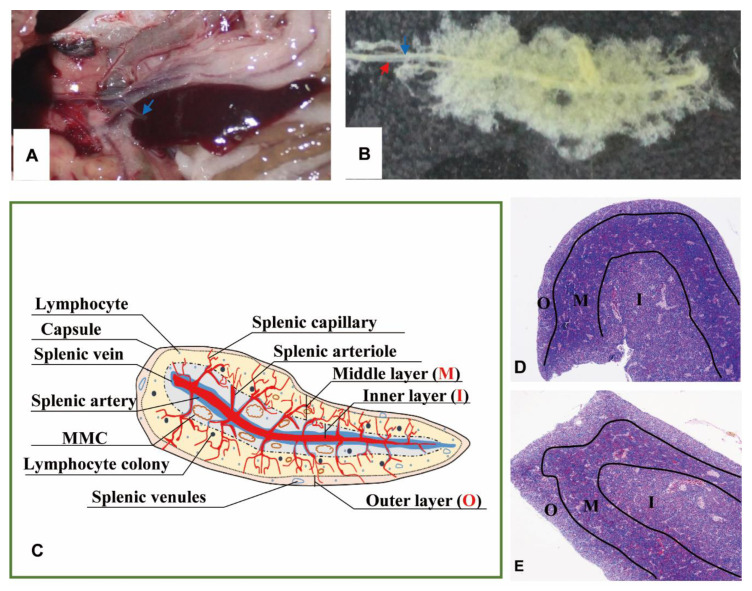
The morphology and portion of the spleen. (**A**). The location of spleen between the gastrointestinal tract, at the head of the spleen sited the splenic portal (→). (**B**). Twenty-four days’ post-corruption with 1% NaOH, showing the splenic artery (→) and splenic vein (→). (**C**). A schematic representation of spleen histological structure. “O”: Outer layer; “M”: Middle layer; “I”: Inner layer; (**D**). Transverse section of the spleen (×40, HE); (**E**). Longitudinal section of the spleen (×40, HE).

The spleen lay in the peritoneal cavity, covered by the liver post-dissected from the left side of the body and was adjacent to the anterior gut wall and the stomach observed from the right side of Nile tilapia (Figure 1A). It was solid, reddish-brown, no accessory, and tongue-shaped with a big round head and a slight end. The absolute weights of spleens were 0.07–0.25 g, and spleen indexes were 0.03–0.14. The splenic portal passed through the head of the spleen and ran parallelly along the splenic longitudinal axis (Figure 1B). 

### 3.2. Micromorphology of the Spleen

#### 3.2.1. Subregions and Components Visualized by H&E Staining

**Figure 2:**
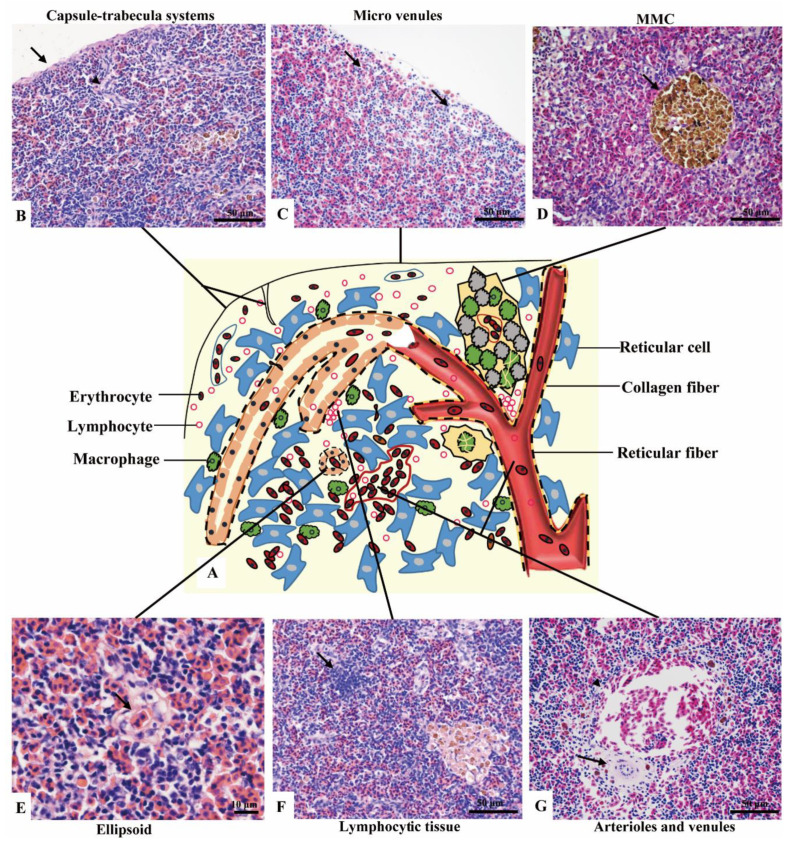
The components of the spleen (H&E staining). (**A**). A schematic representation of spleen; (**B**). The splenic capsule-trabecula systems, showing thin capsule (→) and poorly developed trabecular (▲); (**C**). The micro venules (→) underneath the capsule, consisted of a signal layer of flat endothelial cells with a capacity of 4–6 erythrocytes; (**D**). The MMC (→) brown-black color, sub-circular shape, and surrounded by a single layer of flat cells; (**E**). The ellipsoid (→) consisted of cuboidal-shaped endothelial cells; (**F**). A small cluster of lymphocytes (→) in the middle layer; (**G**). An arteriole (→) and a venule (▲) of the inner layer.

No boundary existed between red pulps and white pulps under a magnification of 40×. However, three subregions could be distinguished according to the sandwich-colored appearance (light blue-navy blue-light blue). The three subregions were named according to their locations (Figure 1C–E): (1) Outer layer: light blue, closed to the capsule; (2) inner layer: light blue, in the center of the spleen with features of vessels; (3) middle layer: navy blue, sandwiched between the out layer and inner layer, characterized by blue clumps. The components were further observed with a magnification of 400×, and a schematic diagram was painted in Word 2003 (Figure 2).

(1) Outer layer

Capsule and the underneath micro-venules were the main elements of the outer layer. The outmost capsule was about 2.6 ± 0.9 μm thick, composed of a single layer of flat mesothelial cells and a few connective tissues. The trabeculae were only and occasionally observed (Figure 2B). The content of erythrocytes was the least among three layers (Figure 2C), as well as the number of lymphocytes, which distributed scattered. MMCs were barely observed.

(2) Middle layer

Red pulps were abundant and the erythrocytes were the richest among the three sub regions. The specific blue clumps outstanding in this layer under 40× magnification were proved to be the small clusters of lymphocytes (Figure 2F). Scattered lymphocytes were also observed. The ellipsoid, another outstanding feature, was distinguished by the pink-colored central capillary (also known as Ellipsoid capillary, EC) (Figure 2E). Melanomacrophage centers (MMCs) appeared independently (Figure 2D). 

(3) Inner layer

The vessels stood out in this layer, including the splenic artery and vein, arterioles, and venules (Figure 2G). The fused MMCs were another character, which was commonly found around the vessels. Besides, the lymphocytes were scattered throughout the subregion. 

#### 3.2.2. Fibrous Skeleton Visualized by Jame’s Staining

**Figure 3:**
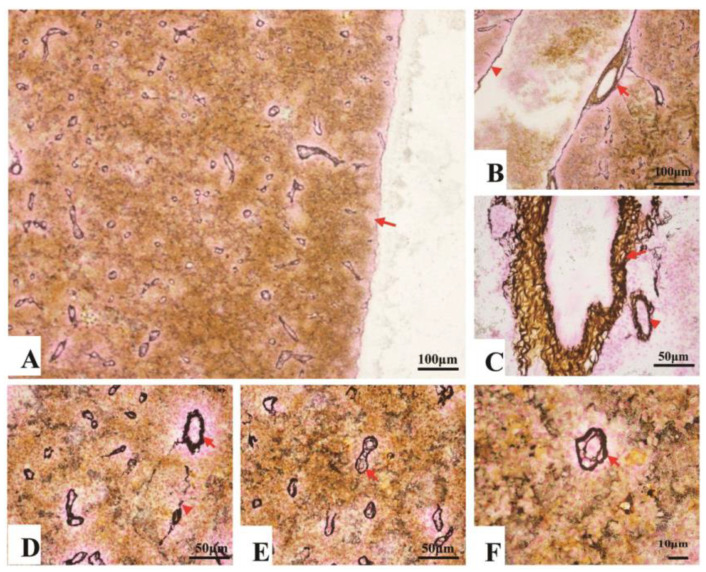
The fibrous skeleton of the spleen (Jame’s staining). (**A**). An overview of the collagen fibers and reticular fibers of the spleen under a magnification of 100×, a thin layer of reticular fibers in the capsule (→); (**B**). Collagen fibers (brown color) and reticular fibers (black color) twisting and surrounding the splenic artery (→) and vein (▲); (**C**). Reticular fibers, circling the splenic arterioles (→) and venules (▲); (**D**). Reticular fibers, circling the splenic arterioles (→) and venules (▲). (**E**) and (**F**). The reticular fibers (→), double layer, forming a concentric circle to sandwich the EC.

Collagen fibers accounted for 7.7–14.3% of the entire splenic area, most of which spread in the splenic cords, and a few of them surrounded the splenic artery and vein (Figure 3A–C). In contrast, reticular fibers accounted for 11.4–18.8% of the entire area, which spread throughout the spleen. About 5.7–9.9% reticular fibers surrounded inside and outside of the EC’s endotheliocytes (Table 1) (Figure 3D–F).

### 3.3. Ultramicroscopic Morphology of Spleen

**Figure 4:**
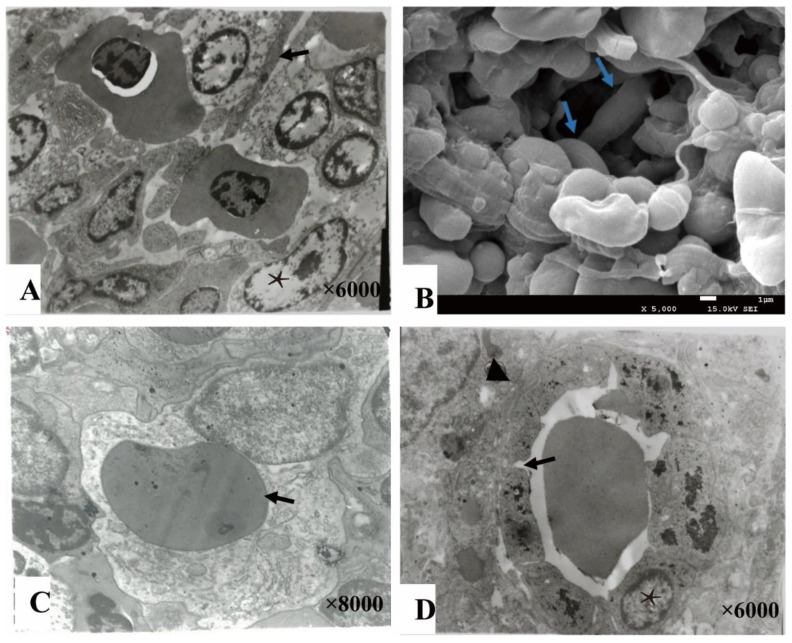
The splenic cord and ellipsoid. (**A**). The reticular cells (★) were adjacent with each other forming net mast allowing deformable erythrocytes (→) to pass through (TME); (**B**). The splenic net, formed by reticular cells allowing the erythrocytes (→) to pass through post deformation (SEM); (**C**). An erythrocyte (→) engulfed by a monocyte cell; (**D**). The ellipsoid consisted of cuboidal-shaped endothelial cells (★) by gap junction (→), and surrounded by fibers (▲).

The splenic cords were mainly composed of reticular cells adjacent to form the splenic net. The erythrocytes passed through the net mesh post-deformation (Figure 4A,B). Close to the net mash monocytes/macrophages and lymphocytes were commonly observed (Figure 4C). A gap about 0.4–1 μm were found between adjacent cells of ellipsoids (Figure 4D).

### 3.4. BSB Visualized by Vital Staining

**Figure 5:**
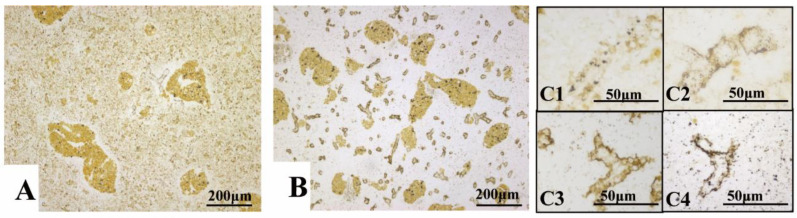

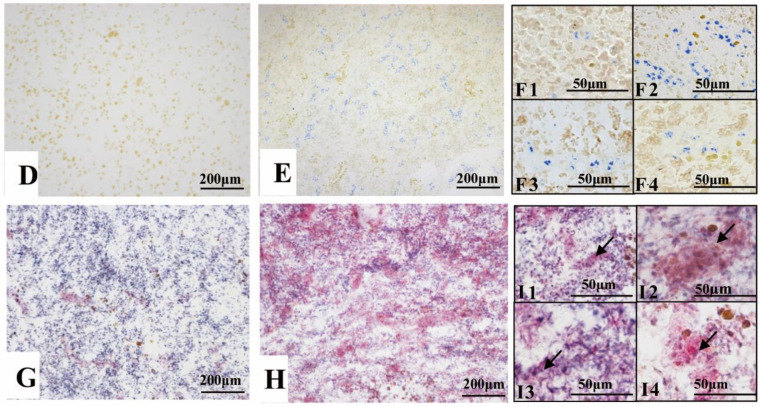
The dynamic monitoring of Trypan blue and ACP (Acid phosphatase) post vital staining. (**A**). 1 hpi (*i.v.*), Trypan blue scattered in the parenchyma and few of them deposited around the ellipsoids; (**B**). 3 hpi (*i.v.*), most of the Trypan blue deposited around the ellipsoids and few appeared in the parenchyma; (**C1**–**C4**)**:** Dynamic changes of Trypan blue deposited around the ellipsoids from 1–24 hpi (*i.v.*), respectively, (**C1**):1 hpi, (**C2**): 2 hpi, (**C3**): 3 hpi and (**C4**): 24 hpi; (**D**): Control spleen without any Trypan blue; (**E**). 8 hpi (*i.p.*), Trypan blue aggregated in the ellipsoids; (**F1**–**F4**): Dynamic changes of Trypan blue in the ellipsoids from 4–72 hpi (*i.p.*), respectively, (**F1**): 4 hpi, (**F2**): 8 hpi, (**F3**): 24 hpi, (**F4**): 72 hpi; (**G**). Normal spleen present little ACP in the endotheliocytes of EC; (**H**). 8 hpi (*i.p.*), ACP appeared in the endotheliocytes of EC and cells of the splenic cords; (**I1**–**I4**): Dynamic changes of the ACP (→) from 4–72 hpi(*i.p.*), respectively, (**I1**): 4 hpi, (**I2**): 8 hpi, (**I3**): 24 hpi, (**I4**): 72 hpi; Staining method: (**A**–**F**), paraffin sections without staining; (**G**–**I**): frozen sections with Gomori staining.

Trypan blue was trapped by the reticular fibers surrounding the ECs. At 1 hpi, the particles were commonly scattered in the splenic parenchyma, whereafter a majority of them were transferred to the reticular fibers around the ECs during the following 2–3 h; 24 h later, the reticular fibers collapsed, resulting in an increase of Trypan blue in the splenic parenchyma (Figure 5A–C). 

**Figure 6:**
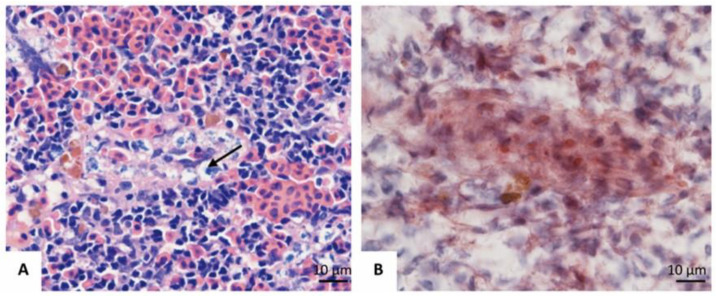

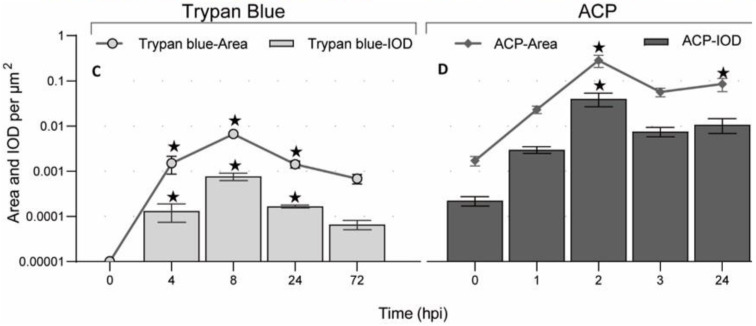
The location and quantification of Trypan blue and ACP (Acid phosphatase). (**A**). 8 hpi (*i.p.*), Trypan blue (→) appeared in the endotheliocytes of ECs; (**B**). 8 hpi (*i.p.*), ACP expressed by the endotheliocytes of ECs; (**C**). Area and IOD of Trypan blue detected using IPP 6.0 from 0–72 hpi (*i.p.*); (**D**). Area and IOD of ACP detected by IPP 6.0 from 0–24 hpi (*i.v.*). “★”: significantly different (*p* < 0.05).

In contrast, Trypan blue was engulfed by the endotheliocytes of ECs post intraperitoneal injection. No particles were observed in the capsule and fibers (Figure 6A). The particles were first observed at 4 hpi, whereafter the area and IOD increased significantly to a summit at 8 hpi. A remarkable decrease of the area and IOD were found at 24 hpi. At 72 hpi, the area and IOD of Trypan blue showed no markable difference to the control group (Figure 5D–F and Figure 6).

ACP was mainly expressed in the endotheliocytes of ECs in normal spleens (Figure 6B). At 4 hpi (*i.p.*), the expression of ACP upregulated slightly judging from the area and IOD, whereafter the area and IOD increased significantly at 8 hpi when both the endotheliocytes of ECs and the cells of the splenic cords expressed ACP. Following 24–72 hpi, the IOD of ACP declined to the normal level, but the area of ACP was significantly broader than that in normal spleens (Figure 5G–I). 

The correlation analysis between the Trypan blue and ACP suggested that the Trypan blue was positively correlated with the ACP (Table S1).

## 4. Discussion

The spleen is a critical organ in immunity of teleost [26]. A larger spleen is regarded as an indirect indicator of immunological status, and reducing the size of the spleen would accelerate the time to death post-challenged [27]. However, the underline mechanisms of the spleen in infection are still ill-defined. To lay basic information for further research, we focused on presenting the splenic structure and the BSB in Nile tilapia. We studied the histology of the spleen and explored the location and function of BSB. We portion the spleen into three subregions according to the sandwiched appearance under 40× magnification. We also found that the BSB existed in the middle layer, composed of the endotheliocytes of EC and the surrounding reticular fibers. Furthermore, the positive correlation between ACP and Trypan blue revealed the clearance function of BSB. Our results will lay the basic information for the mechanism researches, such as the relationship between spleen size and immune, splenic immune process, and pathogenesis.

Usually, the splenic structure was described according to the subregions of the mammals’ spleen, including red pulps, white pulps, marginal region, and capsule [28]. However, in the spleen of teleost red pulps and white pulps are mixed without marginal region, making it difficult to locate each component and compare their differences. According to the sandwiched appearance, we portioned the spleen into three subregions. To our best knowledge, this is the first report about portioning the spleen into three layers by the sandwiched appearance displaying under a magnification of 40×. We described the conventional elements of the spleen in each layer and pointed out the characteristic features of each layer to facilitate recognition. This portion method will guide precise sampling of the ultra-micro specimens after becoming familiar with the location of the components. Furthermore, it could contribute to recognizing the histopathological changes of the spleen post-infection.

The splenic ellipsoid was a unique structure, known as a barrier for filtering and phagocytosis in the chickens [9], soft-shelled turtle (*Pelodiseus sinensis*) [11], Japanese conger (*Conger myriaster*) [29] and darkbarbel catfish (*Pelteobagrus vachelli*) [30]. Consistently, we found that the BSB of Nile tilapia also lay in the ellipsoid, function as a barrier keeping the Trypan blue in the ellipsoid and preventing them from the red pulp. T. Furukawa et al. [29] proved that the ellipsoids filter particles depending on their size. In the present study, Trypan blue was trapped by the ellipsoids, suggesting that the ellipsoids could recognize the size of Trypan blue. Post-injection (*i.v.*), Trypan blue was only confined by the reticular fibers, while the endotheliocytes of EC engulfed the particles when injected through the abdominal cavity. Since no Trypan blue was observed in the capsule of spleen, we suggested that Trypan blue post-intrapeneal injection entered the spleen through blood circulation instead of permeating through the splenic capsule. Therefore, the filter sites of ellipsoids may also depend on the entrance of particles. Besides, carbon, post-injection into chicken and duck of one-day-old, was predominantly found in the extracellular space of the ellipsoids [7], similar to our results that Trypan blue was mainly in the red pulps post-injection (*i.v.*). Therefore, we suggested that reticular fibers in Nile tilapia’s ellipsoids may be as undeveloped as that in a one-day-old duck. 

ACP is an indicator of lysosomes, and it was enhanced during the toxic exposure period or under stress conditions [31,32]. Normally, the splenic clearance of the blood is modest, but when stressed, the clearance capacities will expand [33]. Consistently, we found that a small amount of ACP was expressed in the endotheliocytes of ECs, and the expression of ACP upregulated along with the increase of Trypan blue. Therefore, we indicated that the clearance capacities of endotheliocytes expanded under the stress of Trypan blue. 

## 5. Conclusions

To sum up, the results of vital staining suggested that endotheliocytes and the surrounding reticular fibers composed the BSB, and the former had the capacity of clearance.

## Figures and Tables

**Table 1:** The fibrous skeleton of spleen analyzed by IPP 6.0.

Analysis	A Standard Area (μm^2^)	Area of Fibers (μm^2^)	Percentage
Collagen fibers	275,800	28,876.85 ± 7385.15	7.7–14.3%
Reticular fibers		41,821.99 ± 6948.70	11.4–18.8%
Reticular fibers in ellipsoids		18,002.24 ± 3640.57	5.2–8.8%
Reticular fibers in other areas		23,819.75 ± 4204.20	5.7–9.9%

## Data Availability

All data used in this study are presented in this article.

## Supplementary Materials

The following are available online at https://www.mdpi.com/article/10.3390/ani11102934/s1, Figure S1 Nile Tilapia was dissected from the left side of the body in the order of A–B–C–D, Table S1: Orignal Data of Trypan blue, ACP and fibers used for Statistical analysis in the present study.
