# Supplementary material for: Morphology of the Spleen in Oreochromis niloticus: Splenic Subregions and the Blood-Spleen Barrier
Source: Animals (Basel). 2021 Oct 11;11(10):2934. doi: 10.3390/ani11102934 (PMC8532917; doi:10.3390/ani11102934)
Supplement: Supplementary file 1 [file animals-11-02934-s001.zip › Figure S1.pdf]

Supplementary Materials

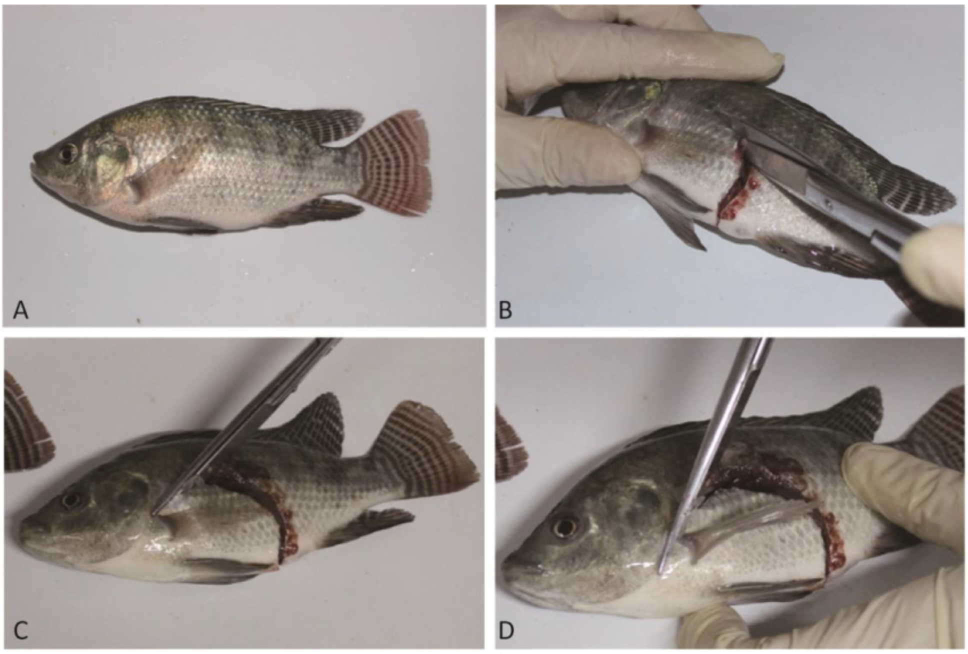

Figure S1 Nile Tilapia was dissected from the left side of the body in the order of A—B—C—D
